# Medical Schools

**Published:** 1845-08

**Authors:** 


### MEDICAL INTELLIGENCE.

Medical Schools.—We have before us the announcements and catalogues of the following medical schools:

*Jefferson Medical College, Philadelphia.* This excellent institution, at its last session, numbered 409 students, and 117 graduates. The annual announcement gives a list of the medical and surgical cases brought before the class during the last session, and by its number and variety, sustains the high reputation of Philadelphia for advantages of clinical instruction. The industry and talent of the Faculty of this institution, have each year approximated more closely the number of their students to that of the oldest institution in the country—the University of Pennsylvania.

*College of Physicians and Surgeons of the City of New York.* The class of last winter, in this institution, numbered 193. Three courses of lectures are annually announced, extending the whole period of public instruction in the College, to eight months. The fall course, by Profs. Smith, Watts, Parker, and Gilman, is free for matriculants to the winter course. It commences on the first Monday of October, and continues during the month. The winter course, as usual, embraces the four months from November 3d to March 1st. The spring course is given in the college, by an association of gentlemen, most of whom are connected with the different public medical institutions in the city. It commences about the middle of March, and continues until June 1st.

*Harvard University Medical Department*, Boston. The class of last year numbered 157. The winter session commences on the first Wednesday of November, and continues four months. A resolution of the medical faculty, defines their relations with other schools as follows:

"That hereafter two full courses of lectures in this school, be required of candidates for the degree of Doctor of Medicine. But for one of these courses, a substitute may be received, in a course of lectures at any other medical institution, in which the number of teachers is not less than six, and in which the time occupied by lectures is not less than four months."

*Albany Medical College.* The catalogue of students embraces 112 names; of graduates, 22. The means of clinical instruction in this institution, as shown by the report of cases and operations before the last class, appear to be ample. The next course commences on the first Tuesday of October, and continues sixteen weeks.

*Medical College of Ohio*, Cincinnati. Notwithstanding the number of rival medical institutions in Ohio, the class at Cincinnati numbered 210, of which 145 were from Ohio. This speaks well for the popularity of the college in its own State. The number of graduates was 47.

*Medical Institute at Louisville.* The class of 1844 and '45 exceeds that of any previous class, not only in this institution, but of any other medical school in the Valley of the Mississippi, numbering 286. The ensuing session commences Nov. 3d, and continues four months. There is also a summer school of medicine connected with the Institute, commencing March 17th, and continuing until the last of October, with a recess during the months of July and August. The Marine Hospital at Louisville affords opportunities of clinical instruction.

*Medical College of South Carolina.*—The class in attendance at the last session, embraced 186. Number of graduates, 74. Clinical instruction is furnished by a hospital attached to the college. The coming session commences on the second Monday of November, and closes on the first Saturday of March.

*Medical College of Louisiana.* Number of students, 93; of graduates, 15. Session commences on the third Monday of November, and continues four months. This institution has been adopted, under the new constitution of the State Convention, as the Medical Department of the University of La.

